# Acknowledgement to Reviewers of *Entropy* in 2018

**DOI:** 10.3390/e21010071

**Published:** 2019-01-15

**Authors:** Entropy Editorial Office

**Affiliations:** MDPI, St. Alban-Anlage 66, 4052 Basel, Switzerland

Rigorous peer-review is the corner-stone of high-quality academic publishing. The editorial team greatly appreciates the reviewers who contributed their knowledge and expertise to the journal’s editorial process over the past 12 months. In 2018, a total of 973 papers were published in the journal, with a median time to first decision of 21.05 days and a median time to publication of 44 days. The editors would like to express their sincere gratitude to the following reviewers for their cooperation and dedication in 2018:
Abah, ObinnaLewis, MarthaAbbaszadeh, SadeghLey, ChristopheAbbes, Ali BenLeyvraz, FrançoisAbbod, MaysamLhotska, LenkaAbdollahzadeh Jamalabadi, Mohammad YaghoubLi, ChengqingAbe, SumiyoshiLi, FangyuAbel, MarkusLi, GaojinAboalayon, KhaldLi, GuanchenAcevedo, Rubén CarlosLi, GuiqiangAcharya, JayadevLi, Kuo-BinAcharya, U. RajendraLi, LinAdachi, KoheiLi, TianchengAdhikari, LasithLi, YantaoAfzal, Muhammad T.Liang, ChaoAgathos, AlexanderLiang, Yeong-CherngAguiar, MarcusLiao, HuchangAguilar, CristinaLicata, IgnazioAguilar, GabrielLiebenehm, SabineAgus, MirianLim, ChjanAhmadi, KavehLim, Ka RamAhmadi, MehdiLima, José LuisAhmed, Mosabber UddinLin, Chiu-YueAimino, RomainLin, ZhipingAitken, StuartLind, HansAkih-Kumgeh, BenLindahl, Jose MerigoAkram, MuhammadLinder, TamásAlagic, GorjanLins, Isis DidierAl-Ashwal, Waddah A.Lio, YuhlongAlbantakis, LarissaLipiński, SewerynAlbash, TameemLips, MarkusAlbu, FelixLisa, BorlandAlbuquerque, RobsonLiška, MarekAlcaine, AlejandroLittle, Douglas J.Alfarano, SimoneLiu, ChangAlhawarat, MohammadLiu, Hung-HuanAliakbarzadeh, MojtabaLiu, JingboAl-Kadi, OmarLiu, QiAllen, J.B.Liu, StephenAlobaid, FalahLiu, YongbinAlodjants, AlexanderLiu, YuxuanAlonso-Serrano, AnaLiu, ZhengweiAltaisky, M.V.Lizier, JosephAmabili, MarcoLlamazares, BonifacioAman, JanLloveras, PolAmigó, José MaríaLluch Lafuente, AlbertoAmoretti, MicheleLo Bosco, Giosue’Amorim, EdgardLo Franco, RosarioAnagnostopoulos, GeorgiosLoaiciga, Hugo A.Ananda, Malwane M.A.Löbel, MartinAnber, Mohamed M.Locatelli, EmanueleAndrade, Luiza H.F.DeLocey, Kenneth J.Anghel, Dragos-VictorLoh, Po-LingAngus, SimonLombardi, OlimpiaAniello, PaoloLombardo, Maria CarmelaAnnamalai, KalyanLong, GuiluAnnila, ArtoLongo, SandroAnselmet, FabienLopes, António M.Antao, Dion S.López De Lacalle, Luis N.Antoni, JeromeLópez, CristóbalAntonopoulos, AngelosLopez, Omar D.Antonucci, AlessandroLópez-Granado, OtonielAntuchevičienė, JurgitaLópez-López, DanielApfelbaum, Evgeny M.Lopez-Monsalvo, Cesar S.Aragoneses, AndrésLoreto, VittorioArampatzis, GeorgiosLoubenets, Elena R.Aranda, MiguelLouzada, FranciscoAraujo, GustavoLouzguine, Dmitri V.Araújo, JoséLowe, RobertAravind, Padmanabhan K.Lu, ChenyangArbuzov, AndrejLu, GuoxiangAretxabaleta, AlfredoLu, ZhixinArndt, MarkusLubchenko, VassiliyAros, RodrigoLuczak-Roesch, MarkusArroyave, RaymundoLuengo, DavidArrue, Begoña C.Luft, AndreasArsenić, IlijaLund, AustinArslan, Suayb S.Lundow, Per HåkanAsgharian, HosseinaliLunin, OlegAshikaga, HiroshiLuongo, OrlandoAsikuzzaman, MdLupo, CosimoAssad, SyedLupo, CosmoAsselmeyer-Maluga, TorstenLyashenko, EugeniaAssis, Francisco Marcos DeLyubushin, AlexeyAtangana, AbdonTahir, M.H. Athenes, ManuelMa, DandanAtman, AllbensMa, DuanchengAtoche, Alejandro CastilloMaboudi, MehdiAttanasi, GiuseppeMacaire, LudovicAusloos, MarcelMaccone, LorenzoAwad, Mohamed M.Macedo, PedroAxinte, EugenMachado, J.A. TenreiroAzami, HamedMachado, PenousalBabu, PonnivalavanMacLean, JasonBacco, DavideMadurga, SergioBachler, MartinMady, Carlos Eduardo KeutenedjianBachmann, MaieMagafas, LykourgosBadescu, ViorelMagda, JulesBadin, GualtieroMago, Pedro J.Bae, YoungchulMahajan, RuhiBahrami, ShahabMahanti, AniketBalaji, BhashyamMahdavi, MahboobeBalakumar, PonnampalamMahian, OmidBalazs, PeterMahmud, ShohelBalduzzi, DavidMaiorino, AngeloBaležentis, TomasMairhofer, LukasBalthazar, José ManoelMaisotsenko, ValeriyBamba, KazuharuMajenz, ChristianBan, Adrian I.Majhi, SubhajitBan, MasashiMakimoto, NaokiBanaitis, AudriusMakinde, Oluwole D.Bandt, ChristophMakowiec, DanutaBanerji, AnindyaMalecha, ZiemowitBang, JeonghoMałecki, KrzysztofBanik, IndranilMalgaretti, PaoloBaras, Alexander S.Malmström, LarsBarbaresco, FredericMalomed, BorisBarbay, SylvainMalyuskin, OleksandrBarbier, Jean François EmmanuelMamageishvili, AkakiBarbieri, GiuseppinaMamdoh Elhalawany, Basembarbosa, jaderManchak, J.B.Barbu, Vlad StefanMancini, StefanoBardera, AntonManis, GeorgiosBardy, ErikMann, AdyBariviera, Aurelio F.Mano, HirokiBariviera, Aurelio FernándezManousiouthakis, VasiliosBarletta, LucaManquinho, VascoBarra, AdrianoMansfield, ShaneBarra, FelipeManta, Vasile I.Barralon, PierreMantzaris, Alexander V.Barranco-Jiménez, MarcoManzanares, José AntonioBarrett, AdamMarchildon, LouisBarsanti, MicheleMarcolli, MatildeBarszczewska-Rybarek, IzabelaMaren, Alianna J.Barton, John P.Mariani, Manuel SebastianBarucca, PaoloMarin, MarinBas, JoanMarinazzo, DanieleBaşağaoğlu, HakanMarkechova, DagmarBasaran, CemalMarkides, ChristosBashirzadeh, YasharMarquet, Pablo A.Basios, VasileiosMarshall, WilliamBasso, VittorioMarsiglietti, ArnaudBasu, AyandranathMaršík, FrantišekBasu, SaikatMarsland, StephenBatista Henriques, IzabelaMarszałek, ZbigniewBatista, Arnaldo GuimarãesMartin, AnthonyBatsidis, ApostolosMartín, DavidBauman, Paul T.Martin, HonorioBaumeler, ÄminMartin, WilliamBaumgartner, John B.Martinez-Jaramillo, SerafinBaxter, GarethMartínez-Rodrigo, ArturoBecattini, FrancescoMartino, Ferdinando DiBecerra, Miguel A.Martins, Ian JamesBecher, VerónicaMartin-Torres, JavierBeck, ChristianMárton, LorincBeckwith, AndrewMartyshev, LeonidBeganovic, NejraMaruta, KazukiBeims, Marcus W.Mary, SébastienBelaïd, AbdelMarzen, SarahBelavkin, RomanMarzolino, UgoBellomo, GuidoMašek, PavelBellos, EvangelosMasetti, GiuseppeBelman-Flores, J.M.Masoumi, MajidBelyaev, EvgenyMassar, SergeBenato, AlbertoMassari, FrancescoBenet, LuisMasse, AntoineBenito, Rosa MaríaMasselli, ClaudiaBen-Menahem, YemimaMassoudi, MehrdadBenzi, RobertoMateo-Lázaro, JesúsBeranek, LadislavMathias, Mauro HugoBerenguel Soria, ManuelMatouk, AhmedBerens, PhilippMatson, Douglas M.Berezny, StefanMatsoukas, ThemisBerezovcki, ArkadiMatsumoto, RyutarohBeris, AntonyMatuszka, TamásBerrett, ThomasMatzen, LauraBerry, TyrusMatzkin, AlexBertin, EricMaudlin, TimBertotti, Maria LetiziaMaunder, RobBérut, AntoineMauro, JohnBhatia, ArchnaMavrovouniotis, MichalisBiagi, Pier FrancescoMayer, AudreyBianco, VincenzoMayer, ChristopherBianucci, MarcoMayo, MichaelBie, RongfangMazurek, PrzemysławBielza, ConchaMazzenga, FrancoBierbrauer, JuergenMazzola, GuglielmoBiferale, LucaMcClay, WilbertBigand, AndréMcGill, CherieBilić, NevenMcGuire, MichaelBilodeau, MichelMcKeague, IanBilotta, EleonoraMedo, MatusBimonte, GiovannaMehner, ThomasBisi, MarziaMehta, DhruvBiswas, SoumyajyotiMei, Shao-HuiBithas, Petros S.Meiburger, Kristen M.Bizarro, João P.S.Melbourne, JamesBjörkman, TorbjörnMeletlidou, EfiBlanco, Manuel JesusMeloni, CarloBlekas, Professor Dr. KonstantinosMendez, JorgeBlesius, LeonhardMendez, Martin OswaldoBloch, MatthieuMéndez, VicençBobillo, FernandoMenendez, HectorBobkov, SergeyMeng, F.Q.Bocharova, Irina E.Mengarelli, AlessandroBöcherer, GeorgMeo, MariannaBodini, AntonellaMercier, DavidBodrova, AnnaMerhav, NeriBognár, Gabriella VadásznéMernik, MarjanBogris, AdonisMerschmann, LuizBoguslavski, KirillMeruane, VivianaBohata, MartinMesa Sanchez, Oscar JoseBohdalová, MáriaMetzler, RalfBojowald, MartinMezei, MihalyBolboaca, Sorana D.Michau, GabrielBolonek-Lasoń, KatarzynaMiettinen, JariBombelli, GiovanniMignan, ArnaudBonnefont, MichelMihai, AdelaBopp, Fritz W.Mihailov, AlexanderBorges, Fernando Da SilvaMihailovic, DragutinBorland, LisaMiklaszewski, AndrzejBorondo, FlorentinoMikulka, JanBorrelli, LuigiMilani, SimoneBos, Joppe W.Milic, VladimirBossé, ÉloiMilionis, AthanasiosBosso, LucianoMilitello, CarmeloBoström, KimMilne, Bruce T.Bosyk, Gustavo M.Milnor, John WillardBottazzi, GiulioMinea, Alina AdrianaBoubchir, LarbiMinussi, Carlos RobertoBoucheron, StéphaneMiranda, Jose Garcia VivasBoudt, KrisMiret-Artés, SalvadorBovenga, FabioMirjalili, SeyedaliBowles, JosephMirza, BilalBowman, CaitlinMisevicius, AlfonsasBoyaval, SébastienMishra, AsitavBoyom, Michel NguiffoMitchison, MarkBranciard, CyrilMix, MichaelBrandão, AlexandreMiyazaki, TakahikoBrandão, Michele A.Mizuno, TakayukiBrandner, KayMolinero, XavierBrassard, GillesMonica, CosteaBravetti, AlessandroMontealegre-Melendez, IsabelBrefeld, UlfMonteiro, JoãoBremer, JörgMontero, ÁlvaroBressan, GianniMontes, IgnacioBridwell, David A.Montessori, AndreaBrieba, Luis G.Montisci, AugustoBrinkworth, RussellMontrésor, SilvioBroadbridge, PhilipMontufar, Guido F.Brostow, WitoldMorabito, Francesco CarloBrown, MichaelMorales Luna, GuillermoBrowne, CameronMora-Rodríguez, JesúsBrunel, NicolasMoraru, LuminiţaBruni, RenatoMoravcik, IgorBruscolini, PierpaoloMoreira Freitas, Ana CristinaBuchmeister, BorutMoreira-Matias, LuisBudkov, Yu. A.Moreno Cuartas, BlancaBudroni, CostantinoMoreno, Juan CarlosBurin, Alexander L.Morgado, Welles Antonio MartinezBurrascano, PietroMorgan, EugeneBuscarino, ArturoMorini, ElenaBusch, ThomasMorison, GordonBustos, Diego MartinMorley, BruceBuszko, KatarzynaMoro, Giorgio J.Butler, JohnMoroni, DavideButun, IsmailMorosuk, TatianaButusov, DenisMorris, James R.Buza, KrisztianMorrison, GregCabral, João M.G.Morrone, PietropaoloCaesarendra, WahyuMoruzzi, Rodrigo BragaCai, BaopingMota, AlziraCai, Yi-FuMota-Babiloni, AdriánCaiado, CamilaMottet, DenisCalado, João M.F.Mousavi, AliCaleffi, MarcelloMousavi, S. MostafaCalero Valdez, AndréMowshowitz, AbbeCampbell, GeoffMueller, DavidCangialosi, DanieleMuhammad, KhanCanivet, YvainMuhammad, NazeerCano, JavierMukherjee, Partha SarathiCanovas Pena, Jose S.Müller, JohannesCao, LiangzhiMüller, VincentCao, ZehongMungan, Carl E.Capecci, ElisaMunoz, Miguel A.Capizzano, Stefano SerraMuramatsu, JunCaplan, JoelMuratore-Ginanneschi, PaoloCaporale, Guglielmo MariaMurcio, RobertoCaraiani, PetreMurillo-Escobar, Miguel AngelCaravelli, FrancescoMurrugarra, DavidCarbone, AnnaMusa, Sarhan M.Carbone, VincenzoMuscato, OrazioCaristi, GiuseppeMusil, JindříchCarletti, VincenzoMussardo, GiuseppeCarlini, MaurizioMustafa, Mustafa A.Carmona, Jose ManuelMuszyński, TomaszCarotenuto, VincenzoMwesigye, AggreyCarravetta, ArmandoMyers, Donald E.Carril, Jose CarbiaMyers, GlennCarrillo-Castrillo, Jesús AntonioMyong, Rho ShinCarroll, SeanNa, Young SangCarter, AlanNadal, Jean-PierreCarvalho, PauloNadolny, ZbigniewCasadio, RobertoNagaoka, HiroshiCasalino, GabriellaNaik, Ganesh R.Casarin, RobertoNakamae, KojiCastaneda, GonzaloNakamula, AtsushiCastellano, Javier GarcíaNakamura, Tadas K.Casteren, Jan VanNalepa, JakubCastiglione, ArcangeloNandi, SaikatCastiglioni, PaoloNanjo, KazuyoshiCastillo Sequera, José LuisNara, ShigetoshiCastro, EnriqueNaranjo, LizbethCaţaron, AngelNarra, SatyanarayanaCaticha, ArielNarusawa, UichiroCattani, CarloNasiriavanaki, MohammadrezaCava, Robert J.Nassar, AntonioCavalcante, Charles CasimiroNasser, RajaiCavallaro, GiuseppeNation, PaulCazabet, RemyNaudts, JanCelledoni, ElenaNavara, MirkoÇengel, Yunus AliNavascues, MiguelCeptureanu, Eduard GabrielNazir, SalmanCeptureanu, Sebastian IonNedjalkov, MihailCercas, FranciscoNelatury, SudarshanCereska, AudriusNelson, KenricCernadas, EvaNemilov, SergeiCerqueti, RoyNemzer, Louis R.Cerulli, GiovanniNene, SaurabhCervigón, RaquelNepomuceno, Erivelton GeraldoÇetin Akinci, TahirNeri, IzaakCetto, Ana MariaNettel, FranciscoChai, LeiNewell, KarlChai, RifaiNewton, AndyChailloux, AndréNguyen, Binh P.Chakrabarty, DaliaNichita, Dan VladimirChakraborti, AnirbanNicholls, GeoffChakraborty, BimanNichols, Jonathan M.Chalishajar, Dimplekumar N.Nicholson, SchuylerChallet, DamienNicolaou, NicolettaChamberlin, RalphNie, QingChamoso, PabloNielsen, JohnChan, K.S.Nieminen, PenttiChancellor, NicholasNieus, Thierry R.Chander, HarishNieuwenhuizen, TheoChang, Yao-TangNiezabitowski, MichałChang, ZhijieNiggemann, OliverChang-Jian, Cai-WanNigmatullin, RamilChapman, RossNikas, George K.Chaturvedi, ItiNikkhah-Moshaie, RoozbehChatzigeorgiou, IoannisNiknam, AlborzChau, Kwok-WingNikolaidis, AthanasiosChau, PhucNikolic, HrvojeChaudhari, PratikNikora, ValdimirChaudhary, UjwalNikulov, AlexeyChellamuthu, Vinodh KumarNilsson, Nils AlbinChen, ChiNimmrichter, StefanChen, ChiachungNino, EliasChen, HaoNiu, ChangningChen, JunNiu, HaiqiangChen, JunxinNivedita, NiveditaChen, LinNkouatchah, Telex Magloire NgatchedChen, NanNo, AlbertChen, Po-NingNobre, FernandoChen, Shin-LiangNocera, FrancescoChen, ShunyunNoga, MarcinChen, Shyi-MingNori, FrancoChen, WenNorsen, TravisChen, YuNorton, JohnChen, ZengqiangNosonovsky, MichaelChen, ZhigangNott, DavidChen, Zhi-GangNouri, NimaCheng, Chi-HoNowakowski, Przemysław RobertCheng, Szu-ChengNowicki, MichałCheng, YongguangNurzaman, SuryaCheng, YongqiangNwankpa, Joseph K.Cheng, ZhiyongObuchi, TomoyukiCheron, GuyOclon, PawelChesneau, XavierOizumi, MasafumiCheung, HowardOjovan, MichaelChi, SienOkniński, AndrzejChiachio, ManuelOksuz, IlkayChiarelli, PieroOKUMURA, SusumuChiavazzo, EliodoroOkuno, RyosukeChicharro, DanielOlbrich, EckehardChichigina, OlgaOldofredi, AndreaChiementin, XavierOliveira, SantiagoChin, Cheng SiongOliveira, Santiago Del RioChinappi, MauroOlmos, Joan GuardiaChincoli, MicheleOmachi, ShinichiroChioar, Ioan-AugustinOmer, Siddig AdamChirikjian, GregoryOneto, LucaChitale, KedarOnken, ArnoChliamovitch, GregorOohama, YasutadaChmelik, FrantisekOprea, IulianaChmiel, WojciechOrd, GarnetChoe, YoonsikOrdonez, GonzaloChomaz, LaurianeOrendáč, MartinChotorlishvili, LevanOriols, XavierChou, Chia-ChunOrlando, DaniloChou, RémiOrović, IrenaChouzenoux, EmilieOrtiz De Zárate, José M.Chovanec, PeterOshan, TaylorChristopoulos, Stavros-RichardO’Shea, TimChrysikopoulos, Constantinos V.Osipov, Grigory V.Ciarleglio, AdamOsman, ShazaliCiccotti, GiovanniOssmann, DanielCicone, AntonioOstoja-Starzewski, MartinCiecholewski, MarcinOsuna-Enciso, ValentínCieslak, JakubOświęcimka, PawełCieśliński, Jan L.Ou, CongjieCimato, StelvioOugolnitsky, GuennadyCimini, GiulioOza, NikunjCinelli, MarcoOzel, OmurCipriani, FabioOzer, EkinCitro, RobertaPachori, Ram BilasCiuonzo, DomenicoPacios, Luis F.Cizek, JanPadmanaban, RajchandarClare, Tami LasseterPaganin, Valdecir AntonioCleaver, GeraldPaige, RobCocconcelli, MarcoPaillusson, FabienCodetta-Raiteri, DanielePaja, WieslawCoecke, BobPaleu, ViorelCohen, AchrafPallotta, GiulianaCohen, EliahuPalm, GüntherCohen, RamiPalma, MassimoColangeli, MatteoPalme, MassimoColhon, MihaelaPalmer, Jason A.Colombi, JohnPamucar, DraganColomer-Llinàs, JoanPan, GuangyuanColominas, Marcelo AlejandroPanacharoensawad, EkaritColonius, FritzPanagiotakis, CostasComin, César HenriquePanão, Miguel R. OliveiraConejero, J. AlbertoPanaousis, ManosConradt, ReinhardPanchal, SatyamConstantinou, AnthonyPankratov, AndreyContreras, IvanPant, JeevanContreras-Reyes, Javier E.Pantazis, GeorgeCopot, CosminPantazis, YannisCorchado Rodríguez, Juan ManuelPanzeri, StefanoCordoba, RicardoPaolucci, MarioCorporal-Lodangco, Irenea L.Pap, GyulaCorreia, LuísPapadakis, GiorgosCorsini, AlessandroPapadimitriou, DimitriosCorso, GilbertoPapadimitriou, IoannisCosma, GeorginaPapadopoulos, BasilCosta, Ana C.S.Papaefthymiou, SpyrosCosta, Manuel Filipe P.C.M.Papaioannou, Vasilios E.Costabile, F.Pappalardo, LucaCosta-Requena, JosePappu, Chandra S.Costello, Ben De LacyParadisi, PaoloCouchot, Jean-FrançoisParaoanu, Gheorghe-SorinCoutelieris, Frank A.Park, Choon-SuCozzolino, LucaPark, JoontaekCremonini, MarcoPark, Ju H.Crepeau, JohnPark, NokeunCrippa, PaoloParks, AllenCristelli, MatthieuParraguez, PedroCrooks, Gavin E.Parrilla, LuisCruz-Hernández, CésarParts, LeopoldCsató, LehelParvan, Alexandru S.Cuadra, LucasParvis, MarcoCudennec, ChristophePassante, RobertoCui, HuijuanPasson, OliverCUI, MiaoPaszto, VitCurado, Evaldo M.F.Patel, SaurinCuriac, DanielPaternostro, MauroCurilef, SergioPati, DebdeepCyklis, PiotrPatriarca, MarcoCzechowski, ZbigniewPatriarca, RiccardoCzippelova, BarboraPaul, Titan C.Czujko, TomaszPaulsen, HaukeD’yachkov, Arkadii G.Paulson, JoelDabiri, ArmanPavelka, MichalD’Abramo, GermanoPavlos, Georgios P.Dafnis, NikolaosPaweł, PławiakDai, WeihuiPecori, RiccardoDaivis, Peter J.Pedersen, TroelsDamaševičius, RobertasPei, ZongruiDa’na, EnshirahPein, FlorianD’Andreagiovanni, FabioPeischl, BernhardDanehkar, AshkbizPejaś, JerzyDanescu, RaduPele, Daniel TraianDaneshkhah, AlirezaPelinovsky, DmitryDaniels, BryanPelt, Daniël M.Dann, MarkusPelusi, DaniloDantan, AurélienPeluso, EmmanueleDapena, AdrianaPeng, GaoliangDarmon, DavidPenkuhn, MathiasDarwen, Paul J.Peppas, Kostas P.Das, ManoharPerc, MatjažDassios, IoannisPerdigão, Rui A.P.Dassios, Ioannis K.Perello, JosepDaub, Eric G.Pérez Zubiaur, PabloDavid, SergioPerez, ArmandoDavis, SergioPérez-Escudero, AlfonsoDay, TroyPérez-García, VicenteDe Andrade Alves, Luiz GustavoPérez-Meana, Héctor ManuelDe Donato, PhilippePerez-Morago, HectorDe Giuli, Maria ElenaPerivolaropoulos, LeandrosDe La Fraga, Luis G.Peshkov, IlyaDe La Madrid, RafaelPesta, MichalDe Leon, ManuelPetcu, DanaDe Meo, PasqualePetrellis, NikosDe Oliveira Junior, SilvioPetrzela, JiriDe Paepe, MichelPevny, TomasDe Palma, GiacomoPiacentini, DanielaDe Sanctis, AngelaPiacentini, FabrizioDEBNATH, SHANTANUPiasecki, KrzysztofDębowski, ŁukaszPica, GiuseppeDedu, SilviaPiccinini, GualtieroDehmer, MatthiasPichl, LukasDeiters, UlrichPięciak, TomaszDel Rio, GabrielPietzonka, PatrickDelgado Prieto, MiguelPigolotti, SimoneDella Rossa, FabioPikovsky, ArkadyDelle Rose, MarcoPiltan, FarzinDellnitz, AndreasPin, PaoloDel-Val-Noguera, ElenaPinheiro, MarioDelvenne, Jean-CharlesPinho, MarceloDemey, LorenzPiqueira, J.R.C.Demirel, YasarPirjan, AlexandruDemirhan, HaydarPirola, CarloDeng, YongPirozzi, EnricaDenisova, NatalyaPisano, RaffaeleDeniziak, StanisławPistone, GiovanniDerakhshani, MaaneliPitas, Charalampos N.Dercole, FabioPitolli, FrancescaDerher, LeePivoluska, MatejDeussen, OliverPlacidi, LucaDeutsch, DavidPlanat, MichelDevine, SeanPlastino, AngeloDewan, AshrafPleimling, MichelDewasme, LaurentPlesinger, FilipD’huys, OttiPljonkin, AntonDi Clemente, RiccardoPlotnitsky, ArkadyDi Corrado, DonatellaPluchino, AlessandroDi Martino, FerdinandoPoddubny, AlexanderDi Mascio, PaolaPodgornik, RudiDi Matteo, AlbertoPogromsky, AlexanderDi Matteo, TizianaPoh, Leong HienDi Nardo, ArmandoPolanski, ArnoldDi Tommaso, DevisPoleto, CristianoDi, HaibinPolettini, MatteoDíaz, IvánPolimeno, AntoninoDick, Thomas E.Polishuk, IlyaDidtlevsen, PeterPolizzotto, CastrenzeDiekman, Amanda B.Poljak, DraganDieks, DennisPollard, BlakeDiep, Hung T.Polyanskii, NikitaDigiesi, SalvatorePoncet, SébastienDimuro, Graçaliz PereiraPooranian, ZahraDing, JintaiPoozesh, SadeghDiniz, Márcio AugustoPop, IoanDionísio, AndreiaPopa, LacramioaraDixit, PurushottamPopelka, StanislavDjeziri, Mohand ArabPopescu, P.G.Dobramysl, UlrichPopkov, Yuri S.Dolfin, MarinaPorwik, PiotrDomanski, Pawel D.Porzio, AlbertoDombek, GrzegorzPosadas-Castillo, CornelioDonato, CafagnaPoulsen, RolfDonelli, MassimoPovstenko, YuriyDong, BoxiangPrasad, V.RamachandraDonner, ReikPrecup, Radu-EmilDonner, Reik V.Preda, VasileDonovan, RichardPręgowska, AgnieszkaDoulamis, NikolaosPreißinger, MarkusDownarowicz, TomaszPressé, SteveDragoi, Elena NiculinaPrieto, Jesús-IgnacioDremin, ViktorPrimak, SergueiDreyer, WolfgangPrior, FredDreżewski, RafałProdanov, DimiterDrikakis, DimitrisProesmans, KarelDroguett, Enrique LópezProukakis, NikolaosDrozdz, StanislawPrusov, EvgenyDruilhet, PierrePryss, RüdigerDuan, JinmingPrzanowski, MaciejDuan, QiangPrzyborski, MarekDubbeldam, JohanPshyk, AleksandrDubenko, IgorPuertas-Centeno, DavidDubey, HarishchandraPugliese, EmanueleDuda, PiotrPujol, Francisco A.Dudczyk, JanuszPumain, DeniseDudina, DinaPusey, Matthew F.Dudina, Dina V.Qi, DiDumic, EmilQi, GuanqiuDuncan, EarlQiao, JunweiDuong, Manh HongQiao, LeiDurejko, TomaszQiao, TongDürr, DetlefQiu, DaowenDuursma, IwanQiu, DongDwivedi, DipankarQuaglio, PietroEbrahimi, AminQuax, RickEckstein, MichałQueirós, SandroEdelman, MarkQueiros, Silvio M. DuarteEfremidze, LevanQuevedo, HernandoEfthymiopoulos, ChristosRadhakrishnan, ChandrashekarEguiraun, HarkaitzRadice, AlessioEhret, UweRae, AlastairEhtemam-Haghighi, ShimaRafał, Marcin LaskowskiEidsvik, JoRagulskis, MinvydasEisemann, MartinRahimi Tabar, Mohammad RezaElara, RajeshRahman, Muhammad TauhidurElasha, FarisRahmat, EllahiEler, Danilo MedeirosRaimbault, JusteEleuch, HichemRajaram, RajeevElfadaly, FadlallaRajković, MilanEllahi, R.Rak, RafałEllerman, DavidRalph, Jason F.Elmore, PaulRamampiaro, HeriEl-Nabulsi, Rami AhmadRamirez, Pedro E.Elorza, IkerRamirez-Reyes, AbdielElze, Hans-ThomasRamirez-Rojas, AlejandroEmary, CliveRamos, Célia M.Q.Emőke, ImreRan, ShijuEmran, Khadijah M.Rana, ShuvenduEnders, PeterRangel, VictorEnescu, DianaRangel-Hernandez, VictorEnßlin, TorstenRanky, Paul G.Enteria, NapoleonRanzato, EliaEntezami, ManiRashidi, Mohammad MehdiEpping, MichaelRass, StefanEriksson, Tobias A.Rasti, PejmanErlandsson, FredrikRatts, EricErmolao, AndreaRauh, JohannesEscamilla Herrera, Lenin FranciscoRaussendorf, RobertEscobar, María-joséRauzy, AtonieEscrig-Olmedo, ElenaRea, WilliamEscudero, JavierReddy, SohailEsfeld, Michael-AndreasReddy, YenumulaEsmaeili, BehzadReeke, George N.Esposito, ChristianReichhardt, CharlesEsposito, MassimilianoReina-Tosina, Luis JavierEstelle, PatriceRemolar Quintana, InmaculadaEtemadpour, RonakRenner, RenatoEtesse, JeanRenza, DiegoEvangelista, Luiz RobertoRequardt, ManfredEvangelista, StefaniaRhi, Seok-HoEvans, MichaelRibeiro, FabianoEvans, Peter W.Ribeiro, Haroldo ValentinEvans, RobinRicci-Tersenghi, FedericoEwing, TimothyRichmond, PeterFabris, DrazenRidolfi, ElenaFacchini, AlessandroRiecan, BeloslavFaccini, FrancescoRiechers, Paul M.Faes, LucaRiegler, Michael AlexanderFaggini, MarisaRifo, LauraFaigl, JanRighi, SimoneFaini, AndreaRigolin, GustavoFalcão, D.S.Rikhtegar, FarhadFalcucci, GiacomoRingbauer, MartinFalk, MartinRini, StefanoFan, Chia-MingRitchey, PhilipFan, PingyiRivera, Francisco F.Fang, LuRivero, Cristian RodriguezFang, YinfengRobbiano, MaríaFarajallah, MousaRobbins, DanielFaria, AlvaroRocchetti, AndreaFarina, LorenzoRocha, JorgeFarouk, AhmedRocha, Luis E.C.Farrokhpanah, AmirsamanRodger, James A.Farsad, NarimanRodger, JenniferFasoli, DiegoRodino, LuigiFavretti, MarcoRodrigo, AlvaroFeckan, MichalRodrigues, Alexandre A.P.Fedorov, AlekseyRodrigues, Maria Manuela FernandesFedotov, AlexanderRodriguez, JavierFelice, DomenicoRodriguez, NibaldoFelisberto, PauloRoga, WojciechFeng, CaiRogers, DavidFeng, YangRohrlich, DanielFera, MarcelloRoli, AndreaFereidountabar, AmirhosseinRomagnoli, AlessandroFernández Vázquez, EstebanRomagnoli, Pierre PaulFernández, AntonioRomeo, JoseFernandez-Gamiz, UnaiRomero, RicFerrara, MassimilianoRominger, Andrew J.Ferrari, MicheleRomito, AlessandroFerraro, ElenaRosas, FernandoFerreira, Fernanda A.Roşca, Alin V.Ferreira, Joao C.Rosenkranz, AndreasFerreira, Joao Carlos AmaroRoso, LuisFerreira, PauloRossi, LucaFerrucci, MassimilianoRosu, HaretFidanova, StefkaRoterman, IrenaFiglus, TomaszRoth, JohannesFilho, Mauro FazionRougier, JonathanFilk, ThomasRoullet, GuillaumeFinamore, Weiler A.Roumy, AlineFink, OlgaRousset, MathiasFinster, FelixRovere, MauroFiori, SimoneRovetta, StefanoFischetti, Massimo V.Rozas-Fernandez, AlbertoFistola, RomanoRúa, Enrique ArgonesFitch, RobertRubi, MiguelFitzner, KrzysztofRubio Alonso, HiginioFlack, RobertRubio, F. JavierFlorea, Olivia AnaRubio, FranciscoFlores, JuanRubio, Jose De JesusFlores-de Santiago, Francisco JavierRucco, MatteoFloría, Luis MarioRuddell, BenjaminFoa Torres, Luis E.F.Rudnicki, ŁukaszFong, Silas L.Ruess, JakobFonollosa, Javier R.Ruggieri, EricForgan, TedRuiz Coll, DamianForti, MauroRumí, RafaelFortuna, LuigiRuppeiner, GeorgeFragassa, CristianoRuppert, LászlóFragos, VassiliosRupšys, PetrasFragoso, RuiRuskin, Heather J.Francesco, FerracutiRuss, MeirFrangopoulos, ChristosRyabko, BorisFrank, MichaelRyabov, ArtemFreitas, AndréRyan, Shawn D.Friák, MartinRybakov, K.I.Friedman, JosephRychkov, ValentinFriedrich, Christoph M.Rychtáriková, RenataFrigo, GuglielmoRyu, Keun HoFriston, KarlRzonca, DariuszFrontistis, ZachariasSaad, WalidFrontoni, EmanueleSabirov, DenisFuchs, GuillaumeSaborido, RubénFujita, HamidoSacré, Pierre-YvesFukuchi, KazutoSadiki, AmsiniFullér, RobertSadowski, ŁukaszFülöp, TamásSadowski, Peter J.Fulton, LawrenceSadre, RaminFurmańczyk, KonradSafaai, HoumanFuron, TeddySaffari Pour, MohsenFuruhata, HitoschiSagar, Robin P.Fuster-Parra, PilarSagheer, AlaaGabarda, SalvadorSahoo, Sujit KumarGabbi, GiampaoloSaid, SalemGabrielli, AndreaSala, MassimilianoGadhoumi, KaisSalamon, PeterGadomski, AdamSalili, Seyyed MuhammadGadouleau, MaximilienSaltas, VassilisGadway, BryceSalvador, ManuelGaggero, TomasoSamsonov, Timofey E.Gaggiotti, OscarSan Miguel, AdrianaGajowniczek, KrzysztofSánchez Lasheras, FernandoGaleano, JavierSanchez, DavidGalhano, AlexandraSánchez, GraciaGallegos-Funes, Francisco J.Sanchez-Taltavull, DanielGamez, DavidSandev, TrifceGams, AndrejSándor, BulcsúGan, ZheSandoval, LeonidasGao, ChangjunSandoval, StevenGao, PeichaoSanjuán, Miguel A.F.Gao, SongSantos, AndrésGao, XiangyunSantos, Jesús DanielGao, YanSantos, RodrigoGao, ZhongkeSantos, RubimGarattini, RemoSanz, Ángel S.García Díaz, PilarSaraiva, Erlandson FerreiraGarcía Kerdan, IvánSarmad, ShokatGarcia-Ayllon Veintimilla, SalvadorSarracino, AlessandroGarcia-Bosque, MiguelSasai, KazutoGarcía-García, ReinaldoSason, IgalGarcía-Herrero, JesúsSaucède, ThomasGarcia-Leon, ManuelSawik, BartoszGarcía-Massó, XavierSbitnev, ValeryGardas, BartłomiejScala, AntonioGardner, AndyScarfone, Antonio M.Garg, LalitScharfenaker, EllisGargano, FrancescoScheicher, Ralph H.Gargano, LuisaSchlitter, JürgenGargiulo, GaetanoSchmandt, BastianGarmatyuk, DmitriySchmelzer, JuernGarrido, JoséSchmidt, Arthur R.Gascon, HugoSchmitt, MichaelGauden, Piotr A.Schnoerr, DavidGaudou, BenoitSchöttle, PascalGaume, EricSchubert, KeithGauvrit, NicolasSchulman, LawrenceGavrielides, TomSchulz, SteffenGavrilik, AlexandreSchumann, AndrewGavrilov, MomčiloSchütze, OliverGAVRILUŢ, Alina CristianaScullion, TomGazeau, Jean-PierreScutari, MarcoGe, HaoSeco, DiegoGedeon, OndrejSedlák, PetrGeiger, BernhardSegall, RichardGeiger, Bernhard C.Segoni, SamueleGelbstein, YanivSekeh, Salimeh YasaeiGelbwaser-Klimovsky, DavidSelby, JohnGencaga, DenizSelle, LaurentGeng, YanlinSelvaprabhu, PoongundranGeorgatis, EmmanuelSelvarajoo, KumarGeorge, SonySengupta, AvikGeorgiev, Danko DimchevSenior, Alistair MGeorgiou, Emmanuel P.Senno, Gabriel IgnacioGerla, BrunellaSeo, Dae-ShikGervino, Gianpiero P.Seo, HwajeongGeva, MotiSerani, AndreaGharbaoui, MolkaSergi, AlessandroGhassemali, EhsanServedio, VitoGhazi-Zahedi, KeyanSessa, SalvatoreGheribi, Aimen. E.Seta, TakeshiGhirardi, GianCarloSfetsos, AthanasiosGholamalizadeh, EhsanShadloo, Mostafa SafdariGhoraani, BehnazShafiee, AfshinGhosh, ArnabShahpar, ShahrokhGhosh, IndranilShaikh, DanishGhosh, SubirShamir, LiorGhosh, SucharitaShan, JuanGiannakis, KonstantinosShang, YilunGiannakopoulos, GeorgeShao, ZhenfengGiedt, JoelSharma, VishalGielen, SteffenSharpee, TatyanaGilbert, AndrewShashidhar, NarasimhaGill, Richard D.Sheikhnejad, YahyaGilles, NottonSheikholeslami, MohsenGimenez-Guzman, Jose ManuelShelley-Tremblay, JohnGinsca, Alexandru LucianShen, ChaoGiorgetti, AndreaShen, JunGiorgi, Gian LucaSherbakov, SergeiGirolami, MarkSheremet, M.A.Gisin, NicolasSheremet, MikhailGissane, ConorShi, JieGiuliani, AlessandroShi, LeiGiuliano, DomenicoShi, XianmingGlabadanidis, Paskalisshi, yongtangGlavatskiy, KirillShibahara, MakotoGlowacz, AdamShihavuddin, ASMGnatyuk, SergiyShikano, YutakaGobattoni, FedericaShilnikov, AndreyGodinez Tello, RichardShin, MinchulGoela, NaveenShmargad, YotamGola, ArkadiuszShojafar, MohammadGoldstein, SheldonShu, HaiGołębiowska, IzabelaShum, KennethGolubev, DmitrySiebesma, PierGomes, Luiz F. Autran M.Silhavy, RadekGómez Aguilar, José FranciscoSilva, Jorge F.Gomez, CarlosSilva, Luiz Eduardo VirgilioGomez, ThomasSimani, SilvioGómez-Aguilar, José FranciscoSimiński, KrzysztofGómez-Andrés, DavidSimon, IstvanGómez-Cuba, FelipeSimpson, LeonieGómez-Déniz, EmilioSingh, NavragGómez-Ruano, Miguel AngelSingh, PrashantGomis-Bresco, JordiSinha, KaushikGonçalves, HernâniSiniscalchi, Sabato MarcoGonçalves, Paulo Jorge SequeiraSivasundaram, SeenithGong, PanŠizling, Arnost L.Gontier, NathalieSizochenko, NataliaGontis, VygintasSkeel, Robert D.González De La Rosa, Juan JoséSkilling, JohnGonzález Ruiz, VicenteSkordas, EfthimiosGonzalez, FernandoSkrzypczyński, PiotrGonzalez-Ayala, JulianSladkowski, JanGonzalo, ConsueloSledge, Isaac J.Goodarzi, MarjanSlusanschi, EmilGoodheart, BenjaminSmall, MichaelGooneie, AliSmarandache, FlorentinGopalakrishnan, SarangŚmieja, MarekGope, ProsantaSmirne, AndreaGorawski, MichalSmith, JimGorban, Alexander N.Smith, Jonathan D.H.Górecki, TomaszSmith, TempleGosse, LaurentSniady, PiotrGoto, Shin-ItiroSoares Júnior, AmílcarGou, JianpingSobrino, Xosé Antón VilaGough, JohnSoekadar, SurjoGoupil, ChristopheSofronov, GeorgyGrabchak, MichaelSohn, Seok SuGradojevic, NikolaSolé, AlbertGrassberger, PeterSoler, Miguel ÁngelGrasso, MatteoSoloveychik, IlyaGray, NicoSoman, RohanGrazian, ClaraSommer, FriedrichGreco, MarcoSong, HoubingGreco, MicheleSong, JunmoGreen, JasonSong, WentuGrendar, MarianSonnino, GiorgioGrigoras, VictorSorguven, EsraGrigoryan, ArtyomSothmann, BjörnGrilli, JacopoSousa, Filipa L.Grima, RamonSousa, João C.Grmela, MiroslavSouto, AndréGrøn, ØyvindSpagnolo, NicolòGrondin, FrancoisSparacino, GiovanniGrooms, IanSparavigna, Amelia CarolinaGrzybowska, KatarzynaSpiliotis, MikeGrzybowski, AndrzejSpüler, MartinGuan, YongSrikanth, R.Guaragnella, CataldoStacey, Blake C.Guariglia, EmanuelStachurski, Z.H.Gudder, StanleyŠtajduhar, IvanGudey, Shyam KumarStamatatos, EfstathiosGüell, OriolStanciu, DorinGuenza, MarinaStandish, RussellGui, WenhaoStankevich, NataliyaGuidi, BarbaraStannett, MikeGuillamon, AntonioStasiak, Michal DominikGuisbiers, GrégoryStaudte, RobertGujrati, PurushottamStavrinides, Stavros G.Gul, FarukStavrou, Photios A.Gulasekaran, RajaguruSteeb, HolgerGunawan, AndreyStefanatos, DionisisGunderov, Dmitriy V.Stefanoiu, RaduGünther, UweStella, AttilioGunton, JamesStemler, ThomasGuo, DezhouStepanov, Nikita D.Guo, JianhuaStepien, KrzysztofGuo, SunŠter, BrankoGuo, YanhuiSterling, MarkGuo, YuzhuSteurer, MiriamGuo, Zeng-YuanStigler, StephenGupta, AbhishekhStinis, PanosGurupur, Varadraj PrabhuStipčević, MarioGutiérrez, Eduardo QuevedoStoehr, JulienGutiérrez-Gutiérrez, JesúsStoyanov, BorislavGuyeux, ChristopheStraccia, UmbertoGuzowska, Malgorzata KlaudiaStramaglia, SebastianoGwak, BogeunStraub, JeremyGwak, JeonghwanStrauss, AlfredGwalani, BharatStrizhak, Pavel A.Haack, GéraldineStrozzi, FernandaHabeck, MichaelStruchtrup, HenningHadi, FatemehStruyve, WardHadley, Mark J.Strzalka, DominikHajdukovic, Dragan SlavkovSTUERGA, DidierHajibagheri, AlirezaŠtys, DaliborHakli, RaulSu, JonathanHall, MichaelSuarez, AlvaroHall, Michael J.W.Subasi, AbdulhamitHalliday, DavidSubasi, YigitHallstedt, BengtŠubelj, LovroHaltmeier, MarkusSuciu, AlinHamann, MichaelSuh, Jin-YooHamed, AliSuhov, YuriHamza, BenSuki, BélaHan, ShaoboSuleman, AbdulHan, WenlinSułowicz, MaciejHan, YongshengSumelka, WojciechHänninen, MariaSun, GuangyuHansen, ClintSun, HuaHaouam, IlyasSun, HuafeiHaq, RizwanulSun, Jia RuiHardal, Ali Ümit CemalSun, KeHarremoës, PeterSun, NingHartich, DavidSun, XiankaiHaruna, TaichiSun, XiaoleiHashizume, YoichiroSun, YongzhengHassan, FirasSundar, V.S.Hatada, RurikoSunkara, VikramHatano, NaomichiSunko, DenisHattori, TomokazuSupek, SelmaHatzivasilis, GeorgeSure, JagadeeshHauptmanns, UlrichSutin, AlexanderHaven, EmmanuelSuweis, SamirHavet, MichelSuzuki, JunHayajneh, ThaierSuzuki, ReijiHayakawa, HisaoSvidzinsky, Anatoly A.Hayakawa, MasashiSvozil, KarlHayashi, FumitakaSwacha, JakubHazan, AurelienSwendsen, Robert H.He, SuiningŚwiderski, BartoszHe, WangpengŚwiderski, WaldemarHe, WeifengSwishchuk, AnatoliyHe, YurongSymko, Orest G.Healy, CorneliusSzabo, LorandHeck, Daniel W.Szajowski, Krzysztof J.Hedayatifar, LeilaSzałowski, KarolHeijungs, ReinoutSzalwinski, ChrisHeinen, MarcoSzavits-Nossan, JurajHell, MartinSzczepanski, JanuszHellings, ChristophSzczypinski, PiotrHemmati, FarzadSze, Raymond Nung-SingHenchman, RichardSzederkényi, GáborHendrix, DavidSzekely, GyorgyHererra, LuisSzeląg, AdamHernaez, MikelSzénási, SándorHernan A., MorenoTacchella, AndreaHernández, AntonioTaddei, FabioHernandez, Jesus C.Tajbakhsh, Shahriar EtemadiHerrera, ManuelTakayasu, MisakoHerrera-May, AgustínTalakoub, OmidHerry, Christophe L.Talbot, DenisHidalgo, ArturoTaler, DawidHidalgo, CesarTamborrino, MassimilianoHidalgo, Cesar A.Tamura, KazuhiroHidetoshi, KonnoTan, VincentHilbert, MartinTanahashi, NorihiroHirai, KeitaTanaka, FuyuhikoHirsch, FlavienTang, SongHitchcock, John M.Tang, YifaHnilo, AlejandroTaniguchi, ShigeruHo Jang, GunTao, JunHochheiser, HarryTapias, DiegoHoel, ErikTarasov, VasilyHofbauer, JosefTargiel, KrzysztofHoffman, K.H.Tărniceriu, DanielaHoffmann, RolfTarpey, ThaddeusHohenwarter, AntonTartaglia, AngeloHölbl, MarkoTassi, AndreaHolik, FedericoTehranipoor, FatemehHolmes, DawnTeitel, StephenHonda, KatsuhiroTeixido, TeresaHong, DaesikTepe, EmreHong, Song-NamTeplyaev, AlexanderHong, Wei-ChiangTerebes, RomulusHönig, VladimírTerpák, JánHorvatić, DavorThakur, Vijay KumarHorwitz, LawrenceThanh, Vo HongHős, CsabaThurner, StefanHosseini, MohsenTian, FuyangHosseinidehaj, NedasadatTibullo, VincenzoHou, Shuhn-ShyurngTilloy, AntoineHoughton, Conor J.Timmermann, DirkHristov, JordanTiozzo, GiulioHron, KarelTlelo-Cuautle, EstebanHsiao, Shih-WenTlili, IskanderHsu, Feng ChiToffano, ZenoHsu, Li-YiToher, Cormac H.Hu, HanToma, AidaHu, QiwenTomasiello, AlessandroHua, ZhongyunTomasz, DurejkoHuang, ChangTomita, Jesuino Takachi​Huang, ​YongxiangTomoyoshi, NishiumuraHuang, XiaTopa, GabrielaHuber, MarcoToporensky, AlekseyHuber, MarcusTorabi, MohsenHuetter, MarkusTornambè, AntonioHuminic, GabrielaTorres, LizethHussain, AkhtarTorres-Sospedra, JoaquínHuynh-The, ThienTort, AdrianoHwang, HeasooTortosa, LeandroHwang, Myung-JoongToschi, NicolaHwang, TzonelihTosic, PredragIana, GabrielTrabs, MathiasIbl, MartinTran, DatIchiki, AkihisaTran, Gia KhanhIde, YusukeTran, Le-NamIgasaki, TomohikoTraversaro, FranciscoIglesias, FélixTravieso, CarlosIglesias, José RobertoTravieso-Gonzalez, Carlos M.Iglói, FerencTravis, CharlesIio, JunTrivellato, BarbaraIkuta, RikizoTsau, Chun-HueiIlg, PatrickTscharner, Vincent VonImre, Attila R.Tsekov, RoumenInamura, KentaroTseng, Chih-YuanInce, RobinTsinaslanidis, ProdromosIndiveri, GiovanniTsinos, ChristosIngber, LesterTsinos, Christos G.Insausti, XabierTsiropoulou, Eirini EleniInuso, GiuseppinaTsoulos, Ioannis G.Ionescu, Valeriu ManuelTsuchiya, YoshishigeIsamu, ShioyaTsugawa, ShoIsidro, Jose MariaTsuruoka, HiroshiIskra, KrzysztofTsuruyama, TatsuakiISLAM, SyedTumulka, RoderichIssler, DieterTunaru, Radu S.Ita, EyoTung, Wen-WenIto, MikioTura I Brugués, JordiIto, ShinyaTura, JordiItoh, MasahikoTurki, SadokIvelić Bradanović, SlavicaTveit, Tor-MartinIversen, Eric JamesUeda, YukiIwakawa, AkiraUffink, JosIzacard, OlivierUhlich, StefanIzquierdo, Eduardo J.Umberto, LuciaIzquierdo, Segismundo S.Unakafov, Anton M.J. Pinho, ArmandoUnakafova, ValentinaJacquir, SabirUohashi, KeikoJaeger, GreggUrbea, Jordi BaroJaeger, MarcUrniezius, RenaldasJafary-Zadeh, MehdiUrquhart, AndrewJahandideh, SamadUruba, VáclavJain, AkankshaUttam, ShikharJakóbczyk, LechUuemaa, EvelynJalalisendi, mohammadV. Ribeiro, HaroldoJalalvand, AzarakhshVaezi, MojtabaJames, RyanVailati, AlbertoJamrozik, ArkadiuszVakkilainen, EsaJamrozik, WojciechValdiserri, PaoloJanovec, JozefValentino, GianlucaJanovec, MichalValiente, DavidJantunen, ErkkiVallejos, RonnyJarray, AbdallahVallianatos, FilipposJärv, LaurValverde, Jose C.Jarzyna, MarcinValverde-Albacete, Francisco J.Jarzynski, ChristopherVan Huffel, SabineJasiul, BartoszVandekerckhove, JoachimJasra, AjayVannitsem, StéphaneJauregui, MaxVanslette, KevinJavaheri Javid, Mohammad AliVarghese Chacko, JenuJaved, Arshad AliVarotsos, PanayiotisJeanne, JamesVasquez, PaulaJeng, YihVazquez-Vilar, GonzaloJensen, Jonas KjærVelkavrh, IgorJeong, Dong HyunVenegas-Andraca, Salvador E.Jessa, MieczyslawVenerus, David C.Jewell, JosephVentouras, ErricosJha, ChandanVera, EstebanJi, JimVerbaarschot, JacobusJi, ShuiwangVerdoolaege, GeertJi, VincentVereshchaka, Alexey A.Jia, DiVerma, RajkumarJia, GaofengVerscharen, DanielJiang, HaoVersteegh, MarijnJiao, JiantaoVezzani, AlessandroJiao, YuboVidiella, AntonioJiménez, FernandoViergever, MaxJin, YanVieru, DumitruJohansson, JimmyVilcu, Gabriel EduardJones, EriitaVilenchik, DanJones, Nick SVilla, AlessandroJong, Gwo-jiaVillalobo, AntonioJordan, AndrewVillecco, FrancescoJose, MarcoVirosztek, DánielJozsa, RichardVisan, TeodorJun, SunghaeVisuri, Ville-ValtteriJun, YonggunVita, Andrea DiJung, Eun SungVitanov, NikolayJung, Young-DaeVitevitch, MichaelJunLin, LinVivo, PierpaoloJurman, GiuseppeVladareanu, LuigeJustin Lars, KirkbyVladeanu, CalinJwo, Dah-JingVlăduțescu, ȘtefanKaced, TarikVluymans, SarahKadir, ShabnamVo, TruongKajanus, MiikaVogel, KristinKakamu, KazuhikoVoigtmann, ThomasKalogeropoulos, NikolaosVolosniev, ArtemKalyagin, ValeryVon Der Linden, WolfgangKambezidis, Harryvon Toussaint, UdoKamsu-Foguem, BernardVourdas, ApostolosKanamori, TakafumiWacker, AndreasKanavos, AndreasWacławczyk, MartaKang, JonghoonWagle, Durgesh V.Kaptay, GeorgeWaldstein, SebastianKarabatsos, GeorgeWallace, DavidKaragrigoriou, AlexWang, CanKarakasidis, TheodorosWang, DongKarakatsanis, L.P.Wang, Gang-JinKarakos, DamianosWang, GuodongKarimipour, ArashWang, HuaKarimov, ArturWang, I-HsiangKaryotis, VasileiosWang, JianqiangKasperski, AndrzejWang, JinKastner, RuthWang, JunKastrup, BernardoWang, LiKateris, DimitriosWang, RosalindKather, Jakob NikolasWang, ShiyanKatina, Polinpapilinho F.Wang, YanKatira, ParagWang, YunKatkov, MikhailWang, ZhangweiKatori, YuichiWartena, ChristianKatsiampa, ParaskeviWatanabe, EiichiKaucic, MassimilianoWatanabe, KazuhoKauranne, TuomoWatanabe, ShunKavic, MichaelWątróbski, JarosławKawamoto, ShoichiWeigel, MartinKazimierczuk, KrzysztofWeinert, FriedelKažys, Rymantas JonasWelch, BrandonKe, Jia-HongWen, BaoleKehrer, TimoWesolowski, KrzysztofKeirsbulck, LaurentWharton, KenKelbert, MarkWhite Sánchez, Juan AntonioKeller, KarstenWhite, MartinKellman, MichaelWhitney, RobertKelly, James F.Wibral, MichaelKenett, Dror Y.Wieczorek, Piotr ZbigniewKeppens, ArnoWilczak, DanielKermanshah, AmirhassanWilk, GrzegorzKerzel, MatthiasWilliams, Gustavious PaulKeshavamurthy, SrihariWilson, Christopher G.Kestler, HansWilson, DalzielKhalaf, WalaaWilson, James B.Khalid, FarrukhWimberger, SandroKhaliq, JibranWiśniewski, MarekKhan, Muhammad ImranWitczak, MarcinKhandoker, AhsanWitzany, JiříKharrazi, AliWlodarczyk, ZbigniewKhoshmanesh, KhashayarWojcieszak, DawidKhosla, Kiran E.Wolf, GuyKhrennikov, AndreiWolf, StefanKhribi, LotfiWolpert, DavidKiayani, AdnanWong, Ka-ChunKibler, MauriceWongkasem, NantakanKilgore, Brian D.Woo, Jae OhKillingback, Timothy P.Woodbury, Allan D.Kim, CheonshikWootters, WilliamKim, Eun-JinWoźniak, MarcinKim, HarksooWrzesiński, DariuszKim, Hee-SeokWu, ChangshanKim, KisikWu, ChenshengKim, Sang-IlWu, FanKim, SungwonWu, Hao-TianKim, Tai HoonWu, Hsien-TsaiKim, Tai-hoonWu, JianhuaKindu, MengistieWu, JinsongKinoshita, TamotuWu, Jong-WuuKipnis, AlonWu, XiaofengKiran, RaviWu, XiaoqunKiss, Laszlo I.Xiao, BoqiKitagawa, JiroXiao, PengfengKjelstrup, SigneXiao, ShiyanKlaers, JanXie, HongboKlement, Erich PeterXiong, HaoKlika, VaclavXu, JasonKlimenko, AlexanderXu, MingtianKlus, StefanXu, ShuKnee, George C.Xu, XiguangKnuth, KevinXu, ZelinKobayashi, KoichiYablonsky, GregoryKoblischka, Michael RudolfYamada, ToruKoch, TobiasYamagishi, MasaoKocharovsky, VitalyYamano, TakuyaKóczy, László Á.Yan, QifaKoh, JaehanYan, RuqiangKoh, Yee RuiYan, YubinKoivisto, TomiYang, FanKolasiński, PiotrYang, Lee-XiengKolchinsky, ArtemyYang, LipingKolokoltsov, VassiliYang, MaoKolyukhin, DmitriyYang, ShimingKomarasamy, MageshwariYang, WenKomiyama, JunpeiYang, XiangKondratiev, YuriYang, Xiao-JunKononovicius, AleksejusYang, Y.Konorski, JerzyYang, YixinKoprinkova-Hristova, PetiaYang, YongjieKorbel, JanYang, YuanKordov, KrasimirYanguang, ChenKorepanov, AlexeyYanushkevich, Svetlana N.Kosar, AliYasusada, NambuKoshkin, SergiyYe, JunKoski, Jonne V.Yentes, JennaKosloff, RonnieYeom, Dong-hanKosmrlj, AndrejYeom, SeokwonKossowski, TomaszYeom, TaihoKostrzewski, MariuszYi, Sang-HoonKostur, MarcinYi, XinpingKotsiantis, SotirisYin, PenghangKouchaki, SamanehYin, ShenKowalski, Frank V.Yokoyama, ShotaKowalski, GregoryYook, Soon-HyungKozlov, GlebYoon, Hyung-KooKrajewski, JarekYoshida, HiroakiKrakauer, Nir Y.Youk, HyunKralevska, KatinaYu, FengKrämer, ManuelYu, HaoranKrauss, ChristopherYu, KemingKrawczyk, Dorota AnnaYu, LeiKrawec, Walter O.Yu, MeichenKreislova, KaterinaYu, Tzyy LeonKresin, VitalyYu, XiaoquanKroupa, TomášYu, YangKryszkiewicz, PawełYuan, JieKudryashov, BorisYuan, ZhenKuehne, HildeYunusa-Kaltungo, AkiluKuffer, MonikaYurchenko, DaniilKugiumtzis, DimitrisYurchenko, NikitaKukulka, David J.Yusenko, Kirill V.Kumagai, AtsuyaZaccaria, AndreaKumar, DevendraZagouras, AthanassiosKumar, PradeepZahorian, Stephen A.Kundu, SnehasisZaitsev, Dmitry A.Kunert-Graf, JamesZakinyan, ArthurKunjwal, RaviŽalik, Krista RizmanKuo, Po-ChihZamora-Martinez, FranciscoKuok, Sin-ChiZanella, MattiaKurevija, TomislavZarpelão, BrunoKuruoglu, ErcanZavadskas, Edmundas KazimierasKuś, MarekZeleny, MartinKustova, ElenaZelevinsky, VladimirKuznetsov, Nikolay V.Zema, Demetrio AntonioKuznetsov, VitalyZeng, XianhaiKwan, Wing ShunZeni, NicolaKwon, Yung-KeunZenil, HectorKyriazis, MariosZhai, ChaoLa Rocca, DariaZhang, ChuanLabatut, VincentZhang, DiLadra, SusanaZhang, HaijunLahti, PekkaZhang, HaochunLaín, SantiagoZhang, HuaLake, Douglas E.Zhang, JunLambiase, GaetanoZhang, KejiaLambiotte, RenaudZhang, PanLambrou, TryphonZhang, PengLampart, MarekZhang, QichunLand, MartinZhang, XiLandeka Dragičević, TibelaZhang, XianmingLandes, JuergenZhang, XiaohongLandi, PietroZhang, XiaoyuanLange, HolgerZhang, YangLangemeier, MichaelZhang, Yi ChengLanger, AndreasZhang, YongLangley, PhilipZhang, YuLara, AdrianaZhang, YudongLaudisa, FedericoZhang, Yu-RanLaw, BruceZhang, YushuLawrence, JayZhang, ZhedongLazarovici, DustinZhang, Zi-KeLê, Hông VânZhao, DongliangLe, ZhangZhao, JunmingLecian, Orchidea MariaZhao, LihaoLee, Chang-HwanZhao, SichengLee, Il-GuZhao, WeiLee, Jae-WeonZhao, YingLee, JinhyoungZheng, GuanglouLee, JulianZheng, Tong-XingLee, Sang HoonZherebtsov, SergeyLee, Sang-GonZhou, LinLee, UnCheolZhou, MengChuLeeuwen, Jan VanZhou, NanrunLefèvre, EricZhou, XunLeff, Harvey S.Zhou, YongchengLefkidis, GeorgZhu, GuohunLeggio, BrunoZhu, Ka-DiLeibovici, DidierZhu, Qun-XiongLeitner, DavidZhu, ShanLelea, DorinZhu, TaishanLeMay, SteveZhu, YongLengani, DavideZhu, ZhongkuiLenk, PeterZhukovsky, KonstantinLenzi, Ervin K.Ziebert, FalkoLenzi, Ervin KaminskiZiemba, PawełLENZI, GiacomoZienert, TiloLenzi, MarceloZimbardo, GaetanoLeo, MarcoZimborás, ZoltánLeonarduzzi, Roberto FabioZimmer, JohannesLeonski, WieslawZimmermann, RainerLeopardi, AngeloZitkovic, GordanLeordeanu, CatalinZlatanović, IvanLerma, ClaudiaZorzi, MattiaLesage, James P.Zou, YongLesne, AnnickZozor, SteeveLesz, SabinaZufiria, Pedro J.Leung, Carson K.Zurita, GroverLevakova, MarieZuylen, Henk J. VanLeVine, Michael V.Zverev, Vladimir I.Levitan, JacobZwanzig, Silvelyn





